# Association between Self-Reported Academic Performance and Risky Sexual Behavior among Ugandan University Students- A Cross Sectional Study

**DOI:** 10.5539/gjhs.v6n4p183

**Published:** 2014-04-16

**Authors:** Devika Mehra, Emmanuel Kyagaba, Per-Olof Östergren, Anette Agardh

**Affiliations:** 1 Social Medicine and Global Health, Department of Clinical Sciences, Malmö, Lund University, Sweden; 2 Department of Dean of Students, Mbarara University of Science and Technology, Uganda

**Keywords:** academic performance, mental health, Uganda, university students, risky sex

## Abstract

Little is known about the association between self-reported academic performance and risky sexual behaviors and if this differs by gender, among university students. Academic performance can create psychological pressure in young students. Poor academic performance might thus potentially contribute to risky sexual behavior among university students. The aim of this study was to investigate the association between self-reported academic performance and risky sexual behaviors, and whether gender affects this relationship among Ugandan university students. In 2010, 1,954 students participated in a cross-sectional survey, conducted at Mbarara University of Science and Technology in southwestern Uganda (72% response rate). Multivariate logistic regression analysis was used for the analysis. 1,179 (60.3%) students in our study sample reported having debuted sexually. Of these 440 (42.2%) used condoms inconsistently with new sexual partners, and 344 (33.6%) had had multiple sexual partners. We found a statistically significant association between poor academic performance and inconsistent condom use with a new sex partner and this association remained significant even after adjusting for all the potential confounders. There was no such association detected regarding multiple sexual partners. We also found that gender modified the effect of poor academic performance on inconsistent condom use. Females, who were poor academic performers, were found to be at a higher risk of inconsistent condom use than their male counterparts. Interventions should be designed to provide extra support to poor academic performers, which may improve their performance and self-esteem, which in turn might reduce their risky sexual behaviors.

## 1. Introduction

Little attention has been given to the association between poor academic performance and risky sexual behavior among young people in sub-Saharan Africa. Results from previous studies in other settings have indicated such an association (Crissey, 2004; Marteleto, Lam, & Ranchhod, 2008; Sabia & Rees, 2011; Spriggs & Halpern, 2008; Vaez & Laflamme, 2008; Yan et al., 2009).

Examinations of the association between academic performance and sexual behavior in relation to other factors that might mediate this association have mainly focused on high school students (Hardy, Astone, Brooks-Gunn, Shapiro, & Miller, 1998; Scaramella, Conger, Simons, & Whitbeck, 1998) and few studies have targeted university students (Rector. R., 2005; Schvaneveldt PL, 2001). Findings from a prospective study of female youth in the US showed that a low grade point average (GPA) in grade 8 was significantly associated with teenage pregnancy in a follow-up four years later (Scaramella et al., 1998). These findings are supported by another study from the US which showed that adolescents with a low GPA had a higher risk of teenage pregnancy than those with a high GPA who delayed their pregnancy until their 20s (Hardy et al., 1998). In a study of urban adolescents in the US, it was found that low academic skills were often associated with pregnancy, rather than pregnancy causing high school dropouts and a life of poverty (Gordon, 1996). Luster and Small found a relationship between low GPA and sexual risk behaviors (higher number of partners and lower condom use) among high school teenagers in four rural counties in the US (Luster, 1994). Similarly, a longitudinal study from South Africa, concluded that high school students who performed well on exams in 2002 were less likely to be engaged in sexual activities three years later (Marteleto et al., 2008).

Studies regarding the association between academic performance and sexual behavior are rare in sub-Saharan Africa. The prevalence of unwanted teenage pregnancies and sexually transmitted infections (STIs) including HIV/AIDS, are higher in this region than in high income countries. Therefore, it would be of value to examine the possible association between academic performance and sexual behavior in a low income setting.

Uganda has one of the world’s youngest populations, the median age is 15 years, with 56% of the population below 18, and 34% between the ages of 10 and 24 (UBOS, 2002). The introduction of Universal Primary Education (UPE) and Universal Secondary Education (USE) has resulted in an increasing number of young people attending schools and universities (National Council for higher Education, 2010). The developmental challenges associated with early adulthood may be intensified by an academic environment that exerts psychosocial pressure to maintain a good level of academic performance while at the same time to be actively involved in social life.

Previous studies from Uganda have shown that between 50% and 70% of Ugandan university students have had sexual experiences (Agardh, Emmelin, Muriisa, & Ostergren, 2010; EAC/EALP, 2010; Sekirime, Tamale, Lule, & Wabwire-Mangen, 2001). A recent survey conducted at six Ugandan universities showed that 24% of the students have had multiple partners during the last 12 months and 51% reported inconsistent condom use (EAC/EALP, 2010). Thus, the public health challenge represented by sexual risk-taking behavior among university students indicates a need for further study of this phenomenon, especially with regard to sexual behavior which may be related to and influenced by academic performance.

Poor academic performance may lead to risky sexual behavior due to an inability to assess interpersonal situations or due to reduced motivation to pursue one’s studies and more time spent on social activities (Luster, 1994). Alternatively, poor academic performance might create stress, leading to diverse risk taking behavior, including alcohol and drug use as well as unprotected sex (Andrews & Duncan, 1997; Bergen, Martin, Roeger, & Allison, 2005; Cox, Zhang, Johnson, & Bender, 2007). Poor academic performers are more likely than high achievers to skip school, have disciplinary problems and associate with deviant peers and this may create social structures for substance abuse (Cox et al., 2007) and risky sexual practices.

A number of studies have shown associations between self-esteem and academic performance (Filozof, 1998; Ross, 2000). A study of high school students indicated that academic results determine self-esteem, which could influence risk-taking behavior, but this impact differs by gender (Filozof, 1998). Evidence has also shown that men and women differ in how they cope with low self-esteem (Filozof et al., 1998).

The relationship between academic performance and sexual risk taking behavior does not appear to be a direct one, and some degree of reciprocity between these two measures might be expected. The aim of this study is to examine the association between self-reported academic performance and risky sexual behaviors, and especially whether gender affects this relationship. The second aim is to investigate other factors that might mediate this association, such as alcohol consumption and poor mental health.

## 2. Methods

### 2.1 Study Design and Setting

The current study adopted a cross-sectional study design. It was conducted at Mbarara University of Science and Technology (MUST), a public university in the centre of the second largest city in Uganda. It is situated in the southwestern part of the country, approximately 350 kms away from the capital city, Kampala. Our study population was undergraduate students of the university from the four faculties of: Science, Medicine, Computer Science, and Development Studies. The sample consisted of 1,954 student participants (72%) out of a total enrolment of 2,706. Since the outcome was risky sexual behavior, the data analysis was based on a subset of 1,179 students who indicated they had debuted sexually. Of the respondents, 58.8% were male (n = 693) and 41.2% female (n = 486). Ethics approval for this project was granted by the Institutional Review Committee at MUST.

### 2.2 Data Collection and Analysis

The entire undergraduate student body of MUST was invited to take part in the survey. A consent form describing the purpose of the study was signed by those who agreed to participate. The research team explained that participation in the study was completely voluntary and anonymity would be guaranteed. The self-administered questionnaires were disseminated in the lecture halls. The contact details of the project’s principal investigator and research assistant were provided in case students had any personal questions. The signed consent forms and completed questionnaires were deposited by each student in a sealed box.

The self-administrated questionnaire comprised of 132 questions on socio-demographic factors, academic performance, social capital, mental health, sexual behavior, alcohol consumption, and other life style factors. As a preliminary step in preparing of the questionnaire a pilot study had been conducted among MUST students through focus group discussions (Agardh, Östergren, & Liljestrand, 2004). The current questionnaire was also used in previous studies of university students in this setting (Agardh, Cantor-Graae, & Ostergren, 2012; Agardh et al., 2010; Agardh, Tumwine, Asamoah, & Cantor-Graae, 2012).

### 2.3 Definition of Variables

#### 2.3.1 Background Variables

Age was dichotomized into ≤ 23 (‘younger’) and > 23 (‘older’). The cut-off was based on the median age of the study sample and was used to examine the extent to which sexual behavior might differ with increasing age.

Area of growing up was categorised in the questionnaire as rural, urban and peri-urban, or small town. The variable was then dichotomised into ‘rural’ or ‘urban’, the latter combining urban, peri-urban and small town.”

Educational level of head of the household during childhood was categorized as ‘did not complete primary school’ and ‘completed primary school’, which were coded as ≤ Primary education and > Primary education.

Age at sexual debut was dichotomized into ≥ 16 years and < 16 years.

#### 2.3.2 Academic Achievement Variables

Academic performance was based on the question ‘How well are your studies going?’ The following alternative responses could be chosen: ‘My studies are going on well; My studies are going reasonably well; My studies are not going as well as I wish’ The variable was then dichotomized so that the first two options were coded as ‘good academic performance’ and the last as ‘poor academic performance’.

Academic year was determined by the question: ‘In what year are you currently?’ The variable was dichotomized into ‘1^st^ year’ and > 1^st^ year’ for those in years 2 to 5.

#### 2.3.3 Social Capital Variables

Trust in others was measured on the basis of answers to questions that have been widely used in empirical studies (Agardh et al., 2010; Giordano & Lindstrom, 2010; Subramanian, Kim, & Kawachi, 2002) : ‘Most people would take advantage of you if they had an opportunity;’ ‘Most people try to be fair’; ‘You can trust most people’; and ‘You cannot be careful enough when dealing with other people.’ The response alternatives were ‘I do not agree at all,’ ‘I do not agree,’ ‘I agree,’ or ‘I agree completely’ and were accordingly assigned values from 1 to 4, with a maximum total score of 16. On the basis of the median score, the variable was categorized into ‘high’ (above the median) and ‘low’ (below the median).

Social participation was defined on the basis of 12 different social activities students may have attended in the previous few months. A validated measure used for this was previously employed in Sweden (Hanson, Ostergren, Elmstahl, Isacsson, & Ranstam, 1997). The variable was dichotomized on the median value: the total scores of those who answered ‘yes’ (maximum total score 12) were coded into ‘high’ (above the median) and ‘low’ (below the median).

#### 2.3.4 Mental Health

The Hopkins Symptom Check List (HSCL-25) was used to assess the mental health of the participants. The scale consists of 15 items analysing symptoms of depression, and 10 items assessing symptoms of anxiety during the week prior to the survey (Derogatis, Lipman, Rickels, Uhlenhuth, & Covi, 1974). In addition, 10 items from the Symptom Checklist 90 (SCL 90) were included to measure symptoms of psychoticism during the past week prior to the survey. Each item was graded on a four point Likert scale (Ventevogel et al., 2007). These scales have been validated and used in different African cultural contexts (Agardh et al., 2012; Lee, Kaaya, Mbwambo, Smith-Fawzi, & Leshabari, 2008).

The total mean mental health scores as well as the mean scores of depression, anxiety, and psychoticism were calculated from the student’s total score for each measure, which was then divided by the number of items for all the responses. The current study utilizes total mental health scores, which were then dichotomized into ‘high score’ and ‘low score’, based on the median split of the distribution of these scores, and is referred in what follows as ‘poor mental health’ and ‘good mental health’, respectively.

#### 2.3.5 Alcohol Consumption Variable

Alcohol consumption on the occasion of sexual intercourse had the following response alternatives: always; almost always; more often than on half of the occasions; about half of the occasions; more seldom than on a quarter of the occasions; almost never and never. The first three options were coded as ‘frequent consumers of alcohol on the occasion of sexual intercourse’ and the last two as ‘infrequent users of alcohol on the occasion of sexual intercourse’.

#### 2.3.6 Sexual Behavior Variables (Dependent Variables)

Multiple sexual partners were ascertained by the response to the question: ‘How many sexual partners have you had during the last 12 months?’ and was dichotomized into ‘0 to1’ and ≤ 2. Students who responded ‘yes’ on the latter option were classified as having multiple sexual partners.

Condom use with new partner was determined by asking ‘How often do you use a condom with a new sexual partner?’ Students who answered ‘always’ were coded as ‘consistent condom use’ and those who chose other alternatives were coded as ‘inconsistent condom use’.

### 2.4 Statistical Analysis

Statistical calculations were carried out using SPSS Statistical Package Version 20.0. Descriptive statistics was used to calculate the prevalence of the variables used in the study. Bivariate logistic regression analysis was performed to calculate the crude odds ratio (OR) with 95% confidence interval (CI), to study the association between socio-demographic factors, self-reported academic performance, alcohol in relation to sexual activity, mental health, and social capital (the predictor variables/covariates) regarding multiple sexual partners and inconsistent condom use (the dependent variables). Multivariate logistic regression was used to control for confounding by stepwise adjustment for sex, age, area of origin, social participation, mental health, alcohol use on the occasion of sexual intercourse, and sexual debut. Estimates of effect modification (synergy) were done as “departure from additivity of effects on the chosen outcome scale” and a calculation of synergy index (SI) was carried out to disclose effect modification between the chosen variables as proposed by Rothman and Greenland (Rothman, Greenland, & Lash, 2008).

The following algorithm was used, whereby SI > 1 signifies a synergistic effect (representing a positive effect modification) and SI < 1 an antagonistic effect (representing a negative effect modification):


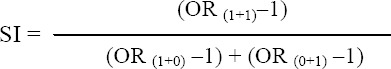


where: OR_(1+1)_ = odds ratio for dummy variable exposed to both factors

OR_(1+0)_ = odds ratio for dummy variable exposed to one factor

OR_(0+1)_ = odds ratio for dummy variable exposed to other factor

OR_(0+0)_ = odds ratio for the dummy variable unexposed to both factors

Significant effect moderation between two variables indicates that when both are present there is an amplified effect, i.e., the combination of the two indicators has a stronger effect, which is higher than added effect of the variables in question.

## 3. Results

Table 1 reports the prevalence of socio-demographic factors, mental health, social capital, sexual behavior, alcohol consumption, and self-reported academic performance among Ugandan university students. Approximately 20% of the students reported poor academic performance. In our sample 42% of the respondents used condoms inconsistently with a new partner. The age of the students was dichotomized into ≤ 23 and > 23 years (65% and 35% respectively). Respondents who were from an urban background totaled 52% and from a rural area 48%. Approximately 30% of the students grew up with a head of the household who had less than a primary education. We observed some gender differences: females had a higher prevalence (50%) of inconsistent condom use than males (37%). There was also a gender difference in the prevalence of students who had multiple sexual partners in the last 12 months (41% for males and 22% for females). The prevalence of alcohol consumption on the latest occasion of sexual intercourse was 16% among males and 9% among females.

**Table 1:** Prevalence of socio-demographic factors, mental health, social capital, sexual behavior, alcohol consumption and self-reported academic performance among Ugandan university students

	All n=1179(%)	Male n=693(%)	Female n=486(%)	X^2^*p*^1^
**Age**				< .001
≤ 23	743 (65.0)	407 (60.5)	336 (71.5)	
> 23	400 (35.0)	266 (39.5)	134 (28.5)	
Missing	(36)	(20)	(16)	
**Area of growing up**				.015
Urban	607 (51.7)	336 (48.8)	271 (56.0)	
Rural	566 (48.3)	353 (51.2)	213 (44.0)	
Missing	(6)	(4)	(2)	
**Educational level of head of household**				.035
> Primary	820 (71.0)	465 (68.6)	355 (74.4)	
≤ Primary	335 (29.0)	213 (31.4)	122 (25.6)	
Missing	(24)	(15)	(9)	
**Mental health**				< .001
Good	524 (47.1)	338 (52.1)	186 (40.1)	
Poor	589 (52.9)	311 (47.9)	278 (59.9)	
Missing	(66)	(44)	(22)	
**Trust in others**				.229
High	639 (60.1)	381 (61.7)	258 (58.0)	
Low	424 (39.9)	237 (38.3)	187 (42.0)	
Missing	(116)	(75)	(41)	
**Social participation**				.036
High	497 (42.2)	310 (44.7)	187 (38.5)	
Low	682 (57.8)	383 (55.3)	299 (61.5)	
Missing	(0)	(0)	(0)	
**Sexual debut**				< .001
≥ 16 years	808 (76.0)	439 (69.4)	369 (85.8)	
< 16 years	255 (24.0)	194 (30.6)	61 (14.2)	
Missing	(116)	(60)	(56)	
**Condom use with a new sexual partner**				< .001
Consistent	603 (57.8)	387 (62.6)	216 (50.8)	
Inconsistent	440 (42.2)	231 (37.4)	209 (49.2)	
Missing	(136)	(75)	(61)	
**Sexual partners in the last 12 months**				< .001
0-1	680 (66.4)	356 (58.7)	324 (77.5)	
> 2	344 (33.6)	250 (41.3)	94 (22.5)	
Missing	(155)	(87)	(68)	
**Alcohol consumption on the occasion of sexual intercourse**				.002
Seldom users	762 (87.2)	433 (84.4)	329 (91.1)	
Frequent users	112 (12.8)	80 (15.6)	32 (8.9)	
Missing	(305)	(180)	(125)	
**Academic year**				.298
1^st^ year	441 (37.6)	250 (36.3)	191 (39.4)	
> 1^st^ year	733 (62.4)	439 (63.7)	294 (60.6)	
Missing	(5)	(4)	(1)	
**Self-reported academic performance**				.416
Good	932 (79.8)	540 (78.9)	392 (81.0)	
Poor	236 (20.2)	144 (21.1)	92 (19.0)	
Missing	(11)	(9)	(2)	

*p*¹ value in table is analyzed based on sex.

Table 2a shows the association between socio-demographic factors, mental health, social capital, sexual behavior, alcohol consumption, and self-reported academic performance with regard to inconsistent condom use with a new partner. Poor academic performance was associated with inconsistent condom use for both males (OR crude 1.59, 95% CI 1.07–2.36) and females (OR crude 2.39, 95% CI 1.44–3.94). Academic year and inconsistent condom use was significantly associated for females (OR crude .67, 95% CI .45–.99) but not for males. Students from a rural background had high odds of inconsistent condom use (OR crude 1.67, 95% CI 1.30–2.14). This finding was similar for males and females. Low social participation was associated with inconsistent condom use with a new partner among males (OR crude 1.89, 95% CI 1.34–2.63). Early sexual debut as well had an association with inconsistent condom use but only for males (OR crude 1.55, 95% CI 1.08–2.22) but not for females.

**Table 2a:** Association (crude odds ratios, 95% Confidence Interval) between socio-demographic factors, mental health, social capital, sexual behavior, alcohol consumption, and self-reported academic performance with inconsistent condom use with a new sexual partner among Ugandan University students

	All n(%)	OR (95 % CI)	Male n(%)	OR (95% CI)	Female n(%)	OR (95% CI)
**Sex**						
Male	231 (37.4)	1 (ref)				
Female	209 (49.2)	1.62 (1.26-2.08)				
**Age**						
>23	64 (36.8)	1 (ref)	45 (34.4)	1 (ref)	19 (44.2)	1 (ref)
≤23	149 (41.5)	1.20 (.92-1.57)	84 (38.4)	1.35 (.96-1.90)	65 (46.4)	.88 (.57-1.35)
**Area of growing up**						
Urban	105 (33.4)	1 (ref)	59 (30.1)	1 (ref)	46 (39.0)	1 (ref)
Rural	113 (48.9)	1.67 (1.30-2.14)	74 (45.1)	1.81 (1.30-2.52)	39 (58.2)	1.67 (1.13-2.47)
**Educational level of the head of the household**						
>Primary education	157 (39.1)	1 (ref)	88 (34.8)	1 (ref)	69 (46.3)	1 (ref)
≤Primary education	59 (44.0)	1.22 (.93-1.60)	44 (43.1)	1.22 (.86-1.72)	15 (46.9)	1.42 (.90-2.23)
**Mental health**						
Good	84 (36.1)	1 (ref)	57 (34.1)	1 (ref)	27 (40.9)	1 (ref)
Poor	122 (43.3)	1.23 (.95-1.58)	71 (41.0)	1.19 (.85-1.66)	51 (46.8)	1.15 (.77-1.70)
**Trust in others**						
High	114 (38.4)	1 (ref)	69 (36.1)	1 (ref)	45 (42.5)	1 (ref)
Low	81 (42.0)	1.04 (.80-1.36)	50 (38.8)	.99 (.69-1.41)	31 (48.4)	1.07 (.72-1.61)
**Social participation**						
High	84 (32.3)	1 (ref)	48 (27.3)	1 (ref)	36 (42.9)	1 (ref)
Low	135 (47.0)	1.59 (1.23-2.04)	86 (46.5)	1.89 (1.34-2.63)	49 (48.0)	1.20 (.81-1.77)
**Alcohol consumed on occasion of sexual intercourse**						
Seldom	289 (41.0)	1 (ref)	74 (35.1)	1 (ref)	62 (47.7)	1 (ref)
Frequent	51 (50.5)	1.47 (.97-2.23)	146 (36.1)	1.54 (.93-2.55)	143 (47.5)	1.71 (.77-3.77)
**Sexual Debut**						
≥16 years	145 (37.7)	1 (ref)	34 (46.6)	1 (ref)	17 (60.7)	1 (ref)
<16 years	58 (42.6)	1.24 (.93-1.67)	48 (41.4)	1.55 (1.08-2.22)	10 (50.0)	1.10 (.63-1.91)
**Academic year**						
1st year	79 (40.1)	1 (ref)	46 (34.3)	1 (ref)	33 (52.4)	1 (ref)
> 1^st^ year	140 (40.2)	.84 (.66-1.09)	88 (38.9)	1.03 (.73-1.45)	52 (42.6)	.67 (.45-.99)
**Self-reported academic performance**						
Good	159 (36.6)	1 (ref)	98 (34.6)	1 (ref)	61 (40.1)	1 (ref)
Poor	59 (54.1)	1.82 (1.34-2.48)	35 (46.1)	1.59 (1.07-2.36)	24 (72.7)	2.39 (1.44-3.94)

Table 2b shows the bivariate associations between socio-demographic factors, mental health, social capital, sexual behavior, alcohol consumption, and self-reported academic performance with regard to multiple sexual partners. The association between self-reported poor academic performance and multiple sexual partners was significant for males (OR crude 1.55, 95% CI 1.04–2.31) but not for females (OR crude .99, 95% CI .55–1.78). Students who were younger than 23 years had a lower risk of having multiple sexual partners (OR crude 0.68, 95% CI 0.52–0.89). Students with poor mental health were found to have a higher likelihood of the same (OR crude 1.45, 95% CI 1.11–1.90). This finding was significant for both males and females. Alcohol consumption on latest occasion of sexual intercourse was significantly associated with multiple sexual partners in males (OR crude 2.94, 95% CI 1.71–5.04) and females (OR crude 5.87, 95% CI 2.49–13.88). Early sexual debut also had a strong association with multiple sexual partners, and this finding was significant for both males (OR crude 2.40, 95% CI 1.67–3.42) and females (OR crude 5.29, 95% CI 2.91–9.56).

**Table 2b:** Association (crude odds ratios, 95% Confidence Interval) between socio-demographic factors, mental health, social capital, sexual behavior, alcohol consumption, and self-reported academic performance with multiple sex partners among Ugandan University students

	All n(%)	OR (95% CI)	Male n(%)	OR (95 % CI)	Female n(%)	OR (95 % CI)
**Sex**						
Female	94 (22.5)	1 (ref)				
Male	250 (41.3)	2.42(1.83-3.20)				
**Age**						
> 23	141 (39.3)	1 (ref)	102 (43.4)	1 (ref)	39(31.5)	1 (ref)
≤ 23	195 (30.6)	.68 (.52-.89)	140 (39.4)	.85 (.61-1.19)	55(19.5)	.53 (.32-2.85)
**Area of growing up**						
Urban	191 (36.0)	1 (ref)	132 (44.6)	1 (ref)	59(25.2)	1 (ref)
Rural	152 (31.1)	.80 (.62-1.04)	117 (38.2)	.77(.56-1.06)	35(19.1)	.70 (.43-1.12)
**Educational level of head of household**						
>Primary education	236 (32.7)	1 (ref)	162 (39.7)	1 (ref)	74(23.6)	1 (ref)
≤Primary education	99 (35.0)	1.11 (.82-1.48)	81 (43.5)	1.17(.82-1.66)	18(18.6)	.74 (.41-1.31)
**Mental health**						
Good	133 (28.7)	1 (ref)	106 (35.1)	1 (ref)	27(16.7)	1 (ref)
Poor	187 (36.9)	1.45 (1.11-1.90)	127 (47.2)	1.65(1.18-2.31)	60(25.2)	1.68 (1.01-2.80)
**Trust in others**						
High	183 (32.9)	1 (ref)	128 (38.8)	1 (ref)	55(24.2)	1 (ref)
Low	121 (32.6)	.98 (.74-1.30)	91 (42.9)	1.19(.84-1.68)	30(18.9)	.72(.44-1.20)
**Social participation**						
High	149 (33.9)	1 (ref)	109 (39.5)	1(ref)	40(24.5)	1 (ref)
Low	195 (33.3)	.97 (.74-1.26)	141 (42.7)	1.14(.83-1.58)	54(21.2)	.82 (.51-1.31)
**Alcohol consumed on the occasion of sexual intercourse**						
Seldom	227 (33.0)	1 (ref)	89 (41.8)	1 (ref)	31(25.0)	1 (ref)
Frequent	62 (66.0)	3.93 (2.49-6.19)	68 (56.7)	2.94(1.71-5.04)	17(43.6)	5.87(2.49-13.88)
**Sexual Debut**						
≥ 16 years	127 (32.9)	1 (ref)	92 (40.0)	1 (ref)	35(22.4)	1 (ref)
< 16 years	87 (64.0)	3.40 (2.51-4.60)	73 (62.4)	2.40(1.67-3.42)	14(73.7)	5.29(2.91-9.56)
**Academic year**						
1st year	116 (30.9)	1 (ref)	82 (37.8)	1 (ref)	34(21.4)	1 (ref)
> 1^st^ year	225 (34.9)	1.20 (.92-1.58)	166 (43.0)	1.24(.88-1.74)	59(22.9)	1.09(.68-1.76)
**Self-reported academic performance**						
Good	261 (32.0)	1 (ref)	186 (38.8)	1 (ref)	75(22.3)	1 (ref)
Poor	78 (38.6)	1.33 (.97-1.84)	60 (49.6)	1.55(1.04-2.31)	18(22.2)	.99(.55-1.78)

Table 3 shows the association between self-reported poor academic performance and inconsistent condom use, which was found to be significant in the fully-adjusted model (Model 7, OR adjusted 1.76, 95% CI 1.19–2.62). By contrast, the association between poor academic performance and multiple sexual partners was significant in the unadjusted model (Model 1). However, when adjusted for poor mental health the association between alcohol consumption on latest occasion of sexual intercourse and early sexual debut became insignificant (OR adjusted 1.20, 95% CI .79–1.82) as shown in (Table 4).

**Table 3:** Association (adjusted odds Ratio, 95% Confidence Interval) between self-reported academic performance and inconsistent condom use with a new partner among Ugandan University students

	Model 1	Model 2	Model 3	Model 4	Model 5	Model 6	Model 7
Poor academic performance	1.99(1.37-2.89)	2.07(1.42-3.01)	1.92(1.31-2.81)	1.88(1.28-2.76)	1.86(1.26-2.75)	1.80 1.21-2.67)	1.76(1.19-2.62)
Female		1.60(1.17-2.17)	1.67(1.21-2.29)	1.61(1.16-2.21)	1.60(1.16-2.12)	1.67(1.20-2.31)	1.78(1.27-2.50)
≤23 years			1.42(1.01-1.98)	1.46(1.04-2.05)	1.46(1.04-2.04)	1.50(1.07-2.11)	1.50(1.06-2.10)
Rural			1.97(1.43-2.71)	1.88(1.36-2.60)	1.88(1.36-2.59)	1.93(1.40-2.67)	1.94(1.40-2.69)
Low social participation				1.58(1.15-2.16)	1.57(1.15-2.16)	1.60(1.14-2.16)	1.56(1.14-2.15)
Poor mental health					1.04 (.75-1.43)	1.00(.72-1.39)	.99(.72-1.37)
Alcohol consumed on the occasion of sexual intercourse						1.67(1.02-2.72)	1.60(.97-2.62)
Early sexual debut							1.38(.95-2.00)

**Table 4:** Association (adjusted Odds Ratio, 95% Confidence Interval) between self–reported academic performance and multiple sex partners among Ugandan University students

	Model 1	Model 2	Model 3	Model 4	Model 5	Model 6	Model 7
Poor academic performance	1.56(1.08-2.27)	1.53(1.05-2.23)	1.64(1.11-2.41)	1.61(1.10-2.37)	1.43 (.96-2.13)	1.31(.87-1.97)	1.20(.79-1.82)
Male		2.21(1.60-3.06)	2.16(1.55-3.02)	2.24(1.60-3.14)	2.48(1.75-3.50)	2.30(1.62-3.27)	1.89(1.31-2.72)
≤23 years			.63 (.46-.88)	.64 (.46-.90)	.61(.44-.85)	.63 (.45-.89)	.61(.43-.87)
Rural			.75 (.54-1.03)	.71 (.52-1.00)	.70(.48-.93)	.70 (.50-.99)	.71(.50-1.01)
Low social participation				1.38(1.00-1.91)	1.36(.98-1.89)	1.36(.98-1.91)	1.37(.97-1.92)
Poor mental health					1.83(1.31-2.57)	1.68(1.19-2.37)	1.65(1.16-2.35)
Alcohol consumed the occasion of sexual intercourse						3.76(2.26-6.27)	3.40(2.02-5.72)
Early sexual debut							2.73(1.86-3.99)

To further investigate the association we used gender as an effect modifier. The analysis showed that the association between poor academic performance and inconsistent condom use was, in fact modified by gender, resulting in an association that was stronger among females (Table 5b). However, gender did not seem to modify the association between poor academic performance and multiple sexual partners.

**Table 5a:** Analysis of effect modification between self-reported academic performance and gender with multiple sexual partners in a sample of Ugandan university students (n = 1179), presented as adjusted Odds Ratios (OR) with 95% Confidence Intervals (CI)

	n (%)	Cases	OR (95 % CI)
Good self-reported academic performance and Male	540 (46.2)	186 (38.8)	1 (Ref)
Good self-reported academic performance and Female	392 (33.6)	75 (22.3)	.45 (.33–.62)
Poor self-reported academic performance and Male	144 (12.3)	60 (49.6)	1.55 (1.04–2.31)
Poor self-reported academic performance and Female	92 (7.9)	18 (22.2)	.45 (.26–.78)
Missing	(11)		

Synergistic index= 0.5.

**Table 5b:** Analysis of effect modification between self-reported academic performance and gender between inconsistent condom use with a new sexual partner in a sample of Ugandan university students (n = 1179), presented as adjusted Odds Ratios (OR) with 95% Confidence Intervals (CI)

	n (%)	Cases	OR (95 % CI)
Good self-reported academic performance and Male	540 (46.2)	169 (35.0)	1 (Ref)
Good self-reported academic performance and Female	392 (33.6)	154 (45.2)	1.53 (1.15–2.03)
Poor self-reported academic performance and Male	144 (12.3)	59 (46.1)	1.59 (1.07–2.36)
Poor self-reported academic performance and Female	92 (7.9)	55 (66.3)	3.65 (2.23–5.97)
Missing	(11)		

Synergistic index= 2.37.

## 4. Discussion

Our study showed that self-reported poor academic performance was associated with inconsistent condom use in both males and females, but this measure did not show any significant association with multiple sexual partners in the last 12 months. We then used gender as an effect modifier and found that females with poor academic performance had a higher risk for inconsistent condom use than men with poor academic performance. However, gender did not seem to modify the effect of poor academic performance on multiple sexual partners.

The association between poor academic performance and multiple sexual partners was significant in the bivariate analysis, but this association disappeared when we adjusted for poor mental health, having consumed alcohol on the latest occasion of sexual intercourse, and early sexual debut. An explanation for this may be that these confounders have a strong association with multiple sexual partners or, alternatively, these measures act as mediating factors in the association. There is evidence from prior research conducted in Uganda and South Africa that indicates these factors are associated with having multiple sexual partners (Lundberg et al., 2011; Zuma et al., 2010). We hypothesize that academic stress results in an unhealthy lifestyle, in which engaging in risky sexual behaviors acts as coping mechanism.

In the total sample, we found a significant association between poor academic performance and inconsistent condom use. This result corresponds to a study conducted in China among undergraduate students, where poor academic performance was one of the risk factors for having multiple sexual partners and inconsistent condom use (Yan et al., 2009). Our findings are also in line with a previous study showing that a low GPA is related to sexual risk-taking behavior (Luster, 1994), is a predictor of early pregnancy (Scaramella et al., 1998), and poor academic performance also leads to more risk- taking with regard to sexual behavior and substance use (Hallfors et al., 2002; Resnick et al., 1997). The last four studies however, were conducted on adolescents rather than on university students and all samples were from high-income countries. Nevertheless, we feel that this association may be even more applicable to those university students, who are poor academic performers and experience a lot of academic stress: they may be less able to apply their cognitive skills, such as risk assessment, with regard to interpersonal situations involving sexual behavior, and one consequence of poor risk assessment might be inconsistent condom use.

In the current study we found that mental health mediated the relationship between poor self-reported academic performance and having multiple sexual partners. Poor mental health and poor academic performance are likely to be inter-related, as poor academic performance can lead to depression, and vice-versa. As a possible psychological compensation for feelings of loss and poor self-esteem, such students may engage in risky sexual activity (Sabia & Rees, 2011). Poor academic performance can lead to considerable stress (Burns, 1991), which may in turn influence mental health.

Alcohol consumption on the latest occasion of sexual intercourse also showed a mediating effect in the association between self-reported academic performance and having multiple sexual partners. It may be that as a result of poor academic performance students turn to high-risk behaviors like alcohol and unsafe sex as ways to reduce their stress. Several studies have shown that alcohol consumption on the occasion of sexual intercourse can lead to unsafe sexual behavior, including the risk of unplanned pregnancies and STIs (Halpern-Felsher, Millstein, & Ellen, 1996; Scott-Sheldon et al., 2012).

In our sample of university students from Uganda the relationship between poor academic performance and risky sexual behavior differed between men and women, particularly with regard to inconsistent condom use. A possible interpretation of the findings showed that academic performance has a greater impact on condom use among women compared to men, may be women perceive themselves lacking the power to negotiate the use of condoms. Women with poor academic performance may perceive that their value as a future partner or spouse has been lowered, and they may feel more unable to demand that the man uses a condom, as compared to women with a good academic record. Although women generally have the power to decide whether a sexual relationship should be initiated, they have less power than men in Uganda with regard to negotiating condom use. Good academic performance, on the other hand, might strengthen women’s self-esteem and feeling of empowerment. With regard to sexual activity, a study of South-African high school students found that those students with good academic performance were less likely to become sexually active (Marteleto et al., 2008). In our study, however, women with poor academic performance were less likely to have multiple sexual partners.

### 4.1 Strength and Limitations

To the best of our knowledge there is very little empirical evidence regarding the association between academic performance and risky sexual behavior among university students in sub-Saharan Africa, and thus the current findings might be important additional knowledge. However, since the study design was cross-sectional, the possibility of causality in both directions should be considered. E.g. it is possible that university students may have moved from a restrictive school environment to a liberal university environment where they are suddenly exposed to parties where alcohol may be involved too, which could lead to unplanned sexual activities. There is evidence in previous research conducted in university students in Uganda and among adolescents in the US which shows that sexual relations at this vulnerable age can lead to concentration problems as it can take their mental resources away from academics which can affect their academic motivation and performance (Gladys, 2013; Sabia, 2007; Sabia & Rees, 2011). According to studies conducted among adolescents in the US, early sexual activity is associated with a higher probability of suspension, unexcused absences from school, a higher likelihood to drop out of school and reduced aspirations to attend university (Rector. R & Johnson. K.A, 2005; Sabia, 2007).

Strength of this study was that it employed self-reported academic performance based on the respondents’ own independent judgment of their academics, irrespective of their examination scores or the perceptions of teachers and parents. A limitation of our study is the cross-sectional design, which does not permit an assessment of the direction of causality between self-reported poor academic performance and risky sexual behaviors. A reverse causation can thus not be ruled out. However, we find it unlikely that reverse causality is the major mechanism behind our findings, as it is difficult to conceive how inconsistent condom use would lead to poor academic performance. It is very likely that risky sexual behavior and poor academic performance are interrelated and thus may be highly reciprocal, so the problems which may develop due to risky sexual behavior (e.g. sexual and reproductive health problems as well as stress) would in turn have a negative impact on academic performance.

Our study results might be generalizable by and large to similar settings in Uganda and other countries in the region, but regional and national trends should also be taken into consideration. With regard to measurement error, there is a possibility of some recall bias concerning the sexual behavior variables, such as number of sexual partners in the past 12 months and the frequency of condom use with a new sexual partner. Moreover, some responses may have been underreported due to the issue of social desirability. We believe that if this were so, it would bias the differences we found towards the null, since it would more likely represent a case of non-differential misclassification than one of differential misclassification. The survey in this study targeted all students at MUST, and had a total response rate of 72%, leaving some room for selection bias. Nevertheless, the reasons for non-participation do not seem to be linked to the main exposures nor to the outcome, since they were mostly due to logistic circumstances or random. In our analyses we adjusted for the most obvious confounding factors, including those that had little impact of the associations we determined. We, therefore, feel that residual confounding would be of minor importance.

## 5. Conclusion

From the results of our current study we can conclude that there is an association between self-reported poor academic performance and risky sexual behaviors among Ugandan university students, although the direction of causality of this association is not clear due to our study’s cross-sectional design. Longitudinal studies would be required to confirm the associations we found. Still the current findings indicate a need to identify students who require extra academic support and to explore their needs for counseling. Such counseling and support may improve their academic performance and self-esteem, which in turn might reduce risky sexual behaviors, alcohol consumption, and drug abuse. We found that female students with poor academic performance seem more vulnerable to the above risks. The universities in Uganda should consider having peer counseling and sex education programs that address safe sexual messaging. This could be achieved through providing knowledge and skills associated with condom use, to prevent unwanted pregnancies and STIs.
